# Enhancing Life-course Analyses of Financial Disadvantage and Depressive Mood Through Longitudinal Design and Causal Inference

**DOI:** 10.2188/jea.JE20250542

**Published:** 2026-05-05

**Authors:** Quan Zhang

**Affiliations:** 1 Department of Quantitative Health Sciences, John A. Burns School of Medicine, University of Hawaiʻi at Mānoa, Honolulu, HI USA; 2 Department of Economics, College of Social Sciences, University of Hawaiʻi at Mānoa, Honolulu, HI USA

To the Editor,

Hiroshi Murayama and colleagues identified five life-course financial-disadvantage trajectories and demonstrated that individuals with increasing affluence reported lower odds of depressive mood than persistently poor peers.^1^ Their use of group-based mixture modelling offers valuable insight into heterogeneity in socio-economic experience. I propose three complementary modules to refine this evidence base and enhance policy translation.

**1. Longitudinal trajectory modelling**

The cross-sectional design and reliance on retrospective socioeconomic status (SES) recall limit causal inference and may introduce misclassification.^1^ A first module would construct dynamic financial-disadvantage trajectories using longitudinal survey waves (eg, the Longitudinal Survey of Middle-Aged and Older Persons) with repeated measures of income, assets, and education. A parameterized cumulative disadvantage index *CFi*(t, w_1_ … w_4_) can weight financial conditions at childhood, early adulthood, midlife, and present, where w_i_ allows sensitivity to different life-stage weights. Latent class growth analysis or group-based multi-trajectory modelling can then identify trajectory classes. Code skeletons using the lcmm or traj packages in R (R Foundation for Statistical Computing, Vienna, Austria) can be shared to facilitate replication. Prospective measurement reduces recall bias^2^ and supports temporally ordered associations. Sensitivity analyses could vary the weights (w_i_) and test alternative numbers of latent classes. I anticipate that longitudinal modelling would attenuate but not eliminate the association between upward mobility and reduced depressive mood and may reveal non-linear patterns, such as late-life declines.

**2. Accounting for unmeasured confounders and mediators**

The original study could not adjust for past depressive episodes, medication use, or comorbid conditions.^1^ A second module would integrate administrative health-claims data to capture antidepressant prescriptions, hospitalizations, and chronic-disease diagnoses, linked to survey respondents via secure identifiers. A propensity-score framework can be implemented with a multinomial-logit model predicting trajectory class membership based on baseline socio-demographics and health history; inverse-probability-of-treatment weights would then adjust logistic regression models of depressive mood. Alternatively, structural-equation modelling or targeted maximum-likelihood estimation could estimate direct and indirect effects of financial trajectories on depression, while incorporating measurement-error models for recall-based SES. Methodological options for purging recall error are discussed in the life-course measurement literature.^3^ By modelling mediators explicitly, investigators may quantify how much of the observed association is explained by comorbidity or prior mental-health status.

**3. Evaluating policy and economic implications**

Upward social mobility has heterogeneous mental-health effects; individuals experiencing downward mobility face elevated depression risk,^4^ while both upward and downward mobility differ from stable high SES.^2^ To inform decision-makers, a third module would simulate the impact of mobility-promoting interventions (eg, cash transfers, re-employment programs) on depressive-mood prevalence and cost-utility outcomes. A Markov model could incorporate transition probabilities between financial-disadvantage states and depressive mood, calibrated from longitudinal data. Parameters include intervention intensity (λ_p_) and effect size on depressive mood (ρ). Using published quality-adjusted life-year weights for depression and Japanese cost data, the model can estimate incremental cost-effectiveness ratios for achieving 1 depression-free year. Monte-Carlo simulation with 10,000 draws would propagate uncertainty. The model could inform whether social policies targeting financial disadvantage are economically justified relative to health-care interventions for depression.

In sum, Murayama et al provide important evidence linking life-course financial mobility to mental health. By adopting prospective cohort designs, employing causal-inference methods to account for confounding and recall bias, and evaluating policy impacts with economic models, subsequent work can strengthen causal understanding and offer actionable guidance for public health and social policy.^1^^–^^5^

## References

[1] Murayama H, Kobayashi E, Sugisawa H, Shaw BA, Liang J. Trajectories of life course financial disadvantage and depressive mood: results from the National Survey of the Japanese Elderly. J Epidemiol. 2026;36(4):132–139. 10.2188/jea.JE20250159 40976701 PMC12975770

[2] Islam S, Jaffee SR. Social mobility and mental health: a systematic review and meta-analysis. Soc Sci Med. 2024;340:116340. 10.1016/j.socscimed.2023.116340 38006845

[3] Chittleborough CR, Baum FE, Taylor AW, Hiller JE. A life-course approach to measuring socioeconomic position in population health surveillance systems. J Epidemiol Community Health. 2006;60(11):981–992. 10.1136/jech.2006.048694 17053288 PMC2465478

[4] Nicklett EJ, Burgard SA. Downward social mobility and major depressive episodes among Latino and Asian-American immigrants to the United States. Am J Epidemiol. 2009;170(6):793–801. 10.1093/aje/kwp192 19671834 PMC2768522

[5] Tampubolon G. Recall error and recall bias in life course epidemiology. Institute for Social Change working paper. 2010. Available at: https://hummedia.manchester.ac.uk/institutes/cmist/archive-publications/working-papers-isc/2010/recallbias-rss-gindo.pdf. Accessed 18.12.2025.

